# Visinin-like protein 1 levels in blood and CSF as emerging markers for Alzheimer’s and other neurodegenerative diseases

**DOI:** 10.1186/s13195-022-01122-4

**Published:** 2022-11-22

**Authors:** Steffen Halbgebauer, Petra Steinacker, Daniel Riedel, Patrick Oeckl, Sarah Anderl-Straub, Jolina Lombardi, Christine A. F. von Arnim, Magdalena Nagl, Armin Giese, Albert C. Ludolph, Markus Otto

**Affiliations:** 1 Department of Neurology, Ulm University Hospital, University of Ulm, Oberer Eselsberg 45, 89081 Ulm, Germany; 2 Deutsches Zentrum für Neurodegenerative Erkrankungen (DZNE e.V.), Ulm, Germany; 3 Department of Neurology, University Clinic, Halle University Hospital, Martin Luther University Halle/Wittenberg, Ernst-Grube Strasse 49, 06120 Halle (Saale), Germany; 4 Division of Geriatrics, University Medical Center Göttingen, Göttingen, Germany; 5 Department of Neuropathology, Ludwig-Maximilians-University, Munich, Germany

**Keywords:** VILIP-1, Alzheimer’s disease, Neurodegeneration, Biomarker, Diagnosis, Prognosis, CSF, Blood

## Abstract

**Background:**

Visinin-like protein 1 (VILIP-1) belongs to the group of emerging biomarkers with the potential to support the early diagnosis of Alzheimer’s disease (AD). However, studies investigating the differential diagnostic potential in cerebrospinal fluid (CSF) are rare and are not available for blood.

**Methods:**

We set up a novel, sensitive single molecule array (Simoa) assay for the detection of VILIP-1 in CSF and serum. In total, paired CSF and serum samples from 234 patients were investigated: 73 AD, 18 behavioral variant frontotemporal dementia (bvFTD), 26 parkinsonian syndromes, 20 amyotrophic lateral sclerosis (ALS), 22 Creutzfeldt-Jakob disease (CJD), and 75 non-neurodegenerative control (Con) patients. The differential diagnostic potential of CSF and serum VILIP-1 was assessed using the receiver operating characteristic curve analysis and findings were compared to core AD biomarkers.

**Results:**

CSF and serum VILIP-1 levels correlated weakly (*r*=0.32 (CI: 0.20–0.43), *p*<0.0001). VILIP-1 concentrations in CSF and serum were elevated in AD compared to Con (*p*<0.0001 and *p*<0.01) and CJD (*p*<0.0001 for CSF and serum), and an increase in CSF was observed already in early AD stages (*p*<0.0001). In the discrimination of AD versus Con, we could demonstrate a strong diagnostic potential for CSF VILIP-1 alone (area under the curve (AUC): 0.87), CSF VILIP-1/CSF Abeta 1-42 (AUC: 0.98), and serum VILIP-1/CSF Abeta 1-42 ratio (AUC: 0.89).

**Conclusions:**

We here report on the successful establishment of a novel Simoa assay for VILIP-1 and illustrate the potential of CSF and serum VILIP-1 in the differential diagnosis of AD with highest levels in CJD.

**Supplementary Information:**

The online version contains supplementary material available at 10.1186/s13195-022-01122-4.

## Background

The analysis of cerebrospinal fluid (CSF) or blood-based biomarkers, reflecting pathophysiologic processes occurring in the central nervous system, might constitute one promising option for the (early) diagnosis of Alzheimer’s disease (AD). For AD, the analysis of amyloid-β peptide 1-42 (Abeta 1-42), total-tau (t-tau), and phosphorylated-tau (p-tau) in CSF is already implemented in the diagnostic guidelines [1]. However, there is a need to develop easy-to-use CSF and especially minimal-invasive blood-based assays using biomarkers reflecting different aspects of the heterogeneous pathology of AD, e.g., synapse loss [2–4], neuroinflammation [5, 6], and disturbed calcium ion homeostasis [7, 8]. One emerging biomarker reflecting the latter is visinin-like protein 1 (VILIP-1). VILIP-1 is a neuronal calcium-sensor protein and a member of the visinin-like protein subfamily [9] expressed in neuronal pericaria, dendrites, and some axons [10]. It is involved in regulating neuronal growth, survival, and synaptic plasticity [11]. AD is associated with a disturbed calcium ion balance [12–14], and calcium-sensor proteins such as VILIP-1 play an important role in the pathology [11, 15]. As disturbed calcium homeostasis causes degeneration of vulnerable neurons and release of VILIP-1 into the extracellular space, VILIP-1 has been rated as a marker of neuronal injury [16–18].

Due to its elevation in the CSF of AD and also in AD patients at a stadium of mild cognitive impairment (AD-MCI), VILIP-1 has been shown to be of diagnostic and prognostic value [19–22]. However, comprehensive studies on VILIP-1 in AD in comparison to other neurodegenerative diseases are rare and studies investigating both CSF and the corresponding blood sample in different neurodegenerative diseases are missing.

Here, we report on the development and validation of a highly sensitive immunoassay for the analysis of VILIP-1 in CSF and blood using single molecule array (Simoa) technology. We applied the novel assay to analyze VILIP-1 concentrations in six different diagnostic groups including AD, behavioral variant frontotemporal dementia (bvFTD), PD spectrum (including Parkinson’s disease (PD), Parkinson’s disease dementia (PDD), and dementia with Lewy bodies (DLB)), amyotrophic lateral sclerosis (ALS), Creutzfeldt-Jakob disease (CJD), and non-neurodegenerative controls (Con). Here, we evaluated the differential diagnostic potential of VILIP-1 CSF and blood levels for neurodegenerative diseases.

## Methods

### Patient selection

All CSF and serum specimens examined in this study were from patients seen in the Departments of Neurology Ulm (between 2010 and 2020) and Göttingen (1997–2003). The study was approved by local Ethics Committees (approval numbers: Ulm 20/10, Göttingen 100305) and conducted following the Declaration of Helsinki. All participants gave their written informed consent to participate in the study.

VILIP-1 was measured in the CSF and serum of 234 patients which were divided into six groups according to their diagnoses: AD, FTD, PD spectrum (PD, PDD, DLB), ALS, CJD, and Con.

The International Working Group 2 criteria were applied for the diagnosis of 73 AD patients [1]. For the diagnosis of bvFTD (*n*=18), the international criteria were used [23, 24]. Twenty-six patients with Parkinson’s syndrome (PD=17, DLB=2, PDD=7) were diagnosed by specialists for movement disorders according to the United Kingdom PD Society Brain Bank criteria [25]. CJD patients (*n*=22) were neuropathologically confirmed cases from the Department of Neurology in Göttingen (unit for transmissible spongiform encephalopathies) [26]. The 20 ALS patients were definite or probable ALS according to the revised El Escorial criteria [27]. All 75 Con patients were non-neurodegenerative controls showing no clinical and radiological signs for neurodegeneration. These patients underwent a lumbar puncture to exclude an acute inflammation of the central nervous system.

Using the clinical dementia rating scale (CDR) [28], the AD group was stratified into AD-dementia and AD-MCI cases. For the classification, the CDR sum of boxes (CDR SOB) was applied [29]. Patients with a CDR SOB score below 2.5 were classified as AD-MCI [30]. PD-MCI diagnosis was made according to consensus criteria proposed by the Movement Disorder Society task force [31]. After stratification of the PD spectrum patients, the group comprises PD patients without cognitive impairment, PD-MCI, and PDD/DLB patients.

### CSF collection and analysis

After lumbar puncture, CSF samples were centrifuged within 30min at 2000 g and supernatant aliquots were frozen in sterile polypropylene tubes at −80°C. Serum was extracted from blood samples by centrifugation (2000 g, 10 min) and stored likewise until analysis.

Quantification of CSF t-tau, p-tau 181, and Abeta 1-42 was performed with commercially available ELISA kits (Fujirebio, Hanover, Germany). VILIP-1 serum and CSF levels were analyzed with a newly established Simoa assay. Intra- and interassay reproducibility was determined by analysis of CSF and serum sample triplicates in three different runs. The lower limit of quantitation (LLOQ) was defined as reported by Andreasson et al. [32]. All measured samples were between the LLOQ and upper LOQ (ULOQ). VILIP-1 was stable for up to at least three freeze and thaw cycles as well as for 5 days at 4°C or room temperature prior to analysis.

### Antibodies and recombinant protein

As capture and detection antibody, a polyclonal antibody specifically recognizing VILIP-1 was used (RD181119100, Biovendor, Brno, Czech Republic). Biotinylation of the detector antibody was performed in a ratio 40:1 (biotin to antibody) according to the protocol provided by Thermo Fisher Scientific (MA, USA). Recombinant VILIP-1 was purchased from LSBio (LS-G610-100, Seattle, USA).

### Novel VILIP-1 Simoa assay for blood and CSF analysis

All Simoa measurements were performed on a fully automated Simoa HD-1 Analyzer (Quanterix, Lexington, USA). Using a coating concentration of 0.2 mg/ml, the capture antibody was coupled to carboxylated paramagnetic beads (Quanterix, MA, USA) according to the protocol of the manufacturer. For the measurement of VILIP-1 in serum and CSF, the number of active beads with coupled antibodies was reduced and replaced by helper beads (Quanterix, Lexington, USA). Approximately 350,000 helper and 150,000 active beads were deployed for measuring one sample. The required amount of beads was washed twice on a magnetic separator with 1% BSA in PBS with 0.05% Tween-20 and was subsequently diluted in this buffer. The biotinylated VILIP-1 detection antibody was diluted in the same buffer to a concentration of 0.5 μg/ml. Streptavidin-β-galactosidase concentrate (SβG) was prepared in SβG-diluent (Quanterix, Lexington, USA) to a final concentration of 25pM. Resorufin-β-D galactopyranoside (Quanterix, Lexington, USA) substrate was used as provided by the manufacturer. Calibrators and 1:4 diluted CSF/serum samples (1% BSA in PBS with 0.05% Tween-20) were transferred into a 96-well plate (Quanterix, Lexington, USA) and placed into the HD-1. For assay configuration, a two-step assay protocol was chosen. In the first step, 25μl of bead and 20μl biotinylated antibody solution were incubated for 30 min (40 cadences) in a reaction cuvette (Quanterix, Lexington, USA) together with the sample, followed by several wash steps. In the second step, 100μl of SβG was added and incubated for 5 min and 15 s (7 cadences), followed by the addition of the substrate and automated imaging. Image analysis was performed by the HD-1 Analyzer software version 1.5 (Quanterix, Lexington, USA). As a regression model, a four-parameter logistic curve with 1/*y* weighting was applied.

### Statistical analysis

For concentration differences between the two groups, the Mann-Whitney *U* test was applied. Kruskal-Wallis test with subsequent Dunn’s post hoc test was applied for comparisons of three or more groups. Cut-off calculations were done with receiver operating characteristic (ROC) analyses. By maximizing the Youden index (sensitivity+specificity−1) and by the selection of the best likelihood ratio, cut-off levels were determined. To assess significant associations between parameters, the Spearman rank correlation coefficient was calculated. For differences in sex distribution, the chi-square test was applied. For all evaluations, *p*<0.05 was considered statistically significant. Calculations were performed using the GraphPad Prism 7.0 software (GraphPad Software, La Jolla, CA, USA).

## Results

### Performance of the newly established VILIP-1 Simoa assay

The Simoa assay covers a range from 0.7 to 1600 pg/ml. The intra-assay CV was 6% for CSF and 7% for serum. The interassay CV for CSF and serum was determined to be 11% and 8%, respectively. Samples were dilution stable between dilutions of 1:2 and 1:10 for CSF and 1:2 and 1:8 for serum. We calculated a LLOQ of 0.8 pg/ml. No cross-reaction with human albumin or immunoglobulin G was observed. More details on the performance of the assay can be found in Additional file 1.

### Clinical and demographic features

In this study, we investigated the CSF and blood of 234 patients comprising 73 AD, 18bvFTD, 26 PD spectrum, 20 ALS, 22 CJD, and 75 non-neurodegenerative control patients. Demographic and clinical parameters of the patient groups are summarized in Table 1. Age and sex did not differ significantly between groups.

**Table 1:** Clinical and demographic features of the diagnostic cohorts

	Con	AD	PD spectrum	bvFTD	ALS	CJD
***N***	75	73	26	18	20	22
**f/m**	45/30	47/26	11/15	9/9	8/12	14/8
**Age (years)**	69±13	70±8	70±8	68±10	64±13	65±8
**CSF VILIP-1 [pg/ml]**^a^	91 (72−119)	154 (119−220)	112 (62−166)	105 (81−134)	98 (64−141)	624 (326−1173)
**Serum VILIP-1 [pg/ml]**^b^	23 (18–31)	33 (24–36)	27 (18–32)	28 (21–42)	26 (20–46)	96 (52–142)
**CSF t-tau [pg/ml]**^c^	239 (159–307)	689 (517–932)	285 (152–325)	331 (260–426)	199 (141–337)	8275 (4403–19,408)
**CSF p-tau 181**	49 (36–70)	120 (94–148)	49 (36–64)	43 (27–57)	37 (29–57)	N/A
**CSF Abeta 1-42 [pg/ml]**^d^	985 (778–1255)	487 (417–593)	899 (517–1116)	871 (735–1157)	945 (786–1147)	N/A
**MMSE**	N/A	23 (17–26)	28 (23.5–29)	26 (24–27)	N/A	N/A
**CDR SOB**	N/A	3.5 (2–5)	0.5 (0–2.5)	3.75 (2.5–8.9)	N/A	N/A

Age in mean±SD. For all other concentrations, the median and IQR are shown. ^a^AD vs. Con and CJD vs. Con, PD spectrum, FTD, ALS *p*<0.0001. AD vs. ALS *p*=0.006, vs. PD spectrum *p*=0.0055, vs. FTD *p*=0.0032, vs. CJD *p*=0.0029. ^b^AD vs. Con *p*=0.0057, CJD vs. Con, vs. AD, vs PD spectrum *p*<0.0001. CJD vs. FTD *p*=0.0002. CJD vs. ALS *p*=0.0004. ^c^AD and CJD vs. all other groups *p*<0.0001. AD vs. CJD *p*=0.033. ^d^AD vs. all other groups *p*<0.0001; AD, Alzheimer’s disease; ALS, amyotrophic lateral sclerosis; CDR SOB, clinical dementia rating sum of boxes; CJD, Creutzfeldt-Jakob disease; Con, non-neurodegenerative control; CSF, cerebrospinal fluid; f, female; FTD, frontotemporal dementia; IQR, interquartile range; m, male; MMSE, Mini-Mental State Examination; N/A, not applicable; PD, Parkinson’s disease

### VILIP-1 concentrations in the different diagnostic groups

**Fig. 1:**
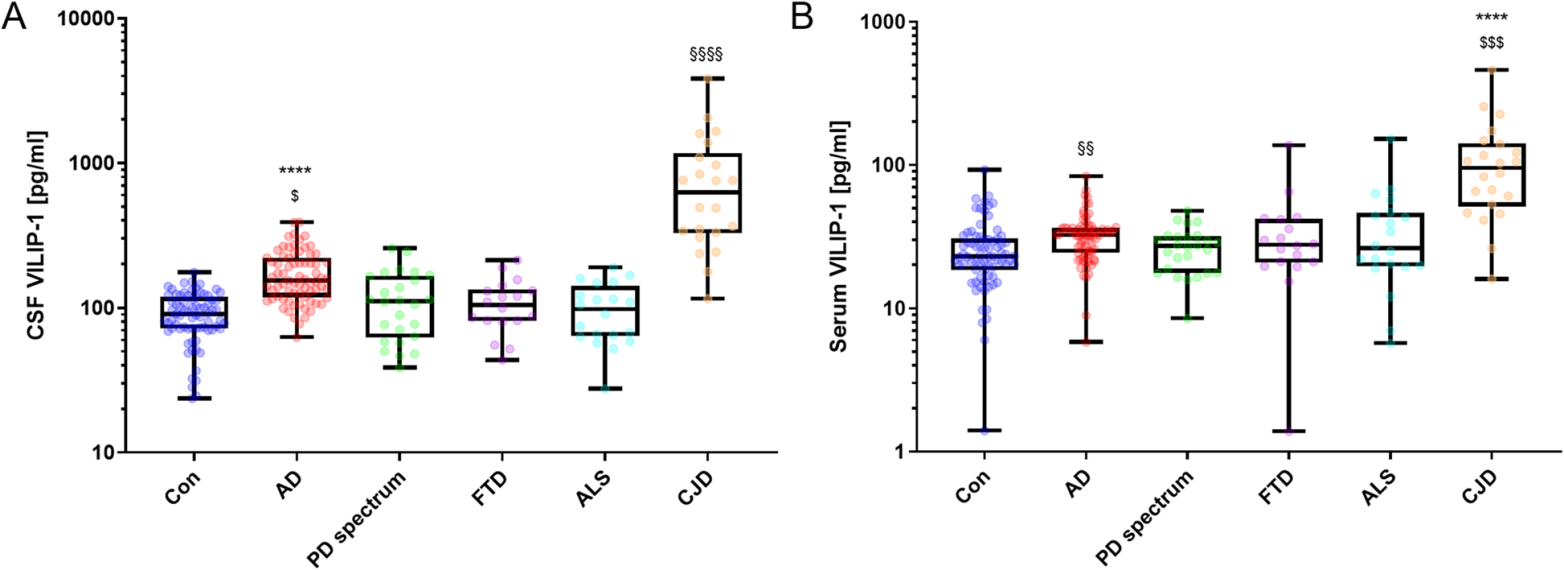
CSF and serum VILIP-1 results in the diagnostic groups. **A** CSF VILIP-1 levels in the diagnostic groups. ****, *p*<0.0001 vs. Con. §§§§, *p*<0.0001 vs. Con, PD spectrum, bvFTD, and ALS. $, *p*=0.0006 vs. ALS, *p*=0.0053 vs. PD spectrum, *p*=0.017 vs. bvFTD, and *p*=0.0026 vs. CJD. **B** Serum VILIP-1 levels in the diagnostic groups. For §§, *p*=0.0055 vs. Con. ****, *p*<0.0001 vs. Con, AD, and PD spectrum. $$$, *p*=0.0005 vs. bvFTD, *p*=0.0004 vs. ALS. For CSF and serum analysis: Con: *n*=75, AD: *n*=73, PD spectrum: *n*=26, bvFTD: *n*=18, ALS: *n*=20, CJD: *n*=22. Concentrations are displayed as box plots with single patients shown. Illustrated are the median concentration, the 25% and 75% percentile, and whiskers from minimum to maximum. Groups were compared by the Kruskal-Wallis test and Dunn’s post hoc test. AD, Alzheimer’s disease; ALS, amyotrophic lateral sclerosis; CJD, Creutzfeldt-Jakob disease; Con, non-neurodegenerative controls; bvFTD, behavioral variant frontotemporal dementia; PD, Parkinson’s disease

CSF VILIP-1 levels in AD were significantly elevated in comparison to Con (*p*<0.0001), PD spectrum (*p*<0.01), bvFTD (*p*<0.01), and ALS (*p*<0.001) (Fig. 1A). Of all the groups analyzed, the highest VILIP-1 values were observed in CJD patients with a significant increase compared to every other patient group (*p*<0.01). Serum VILIP-1 concentrations were elevated in AD compared to Con patients (*p*<0.01). CJD serum levels were significantly increased compared to every other group (*p*<0.001) (Fig. 1B).

### Correlations of CSF and serum VILIP-1 with age, dementia scores, and AD biomarkers

We found no correlation between the age of the patients and CSF (*r*=0.06 (CI: −0.08–0.19), *p*=0.40) as well as serum (*r*=−0.05 (CI: −0.18–0.08), *p*=0.42) VILIP-1 concentrations in the entire cohort. In the Con group alone, age and serum VILIP-1 values also depicted no correlation (*r*=−0.08 (CI: −0.31–0.16), *p*=0.44) whereas age and CSF levels were correlating significantly (*r*=0.33 (CI: 0.11–0.53), *p*<0.01).

**Fig. 2:**
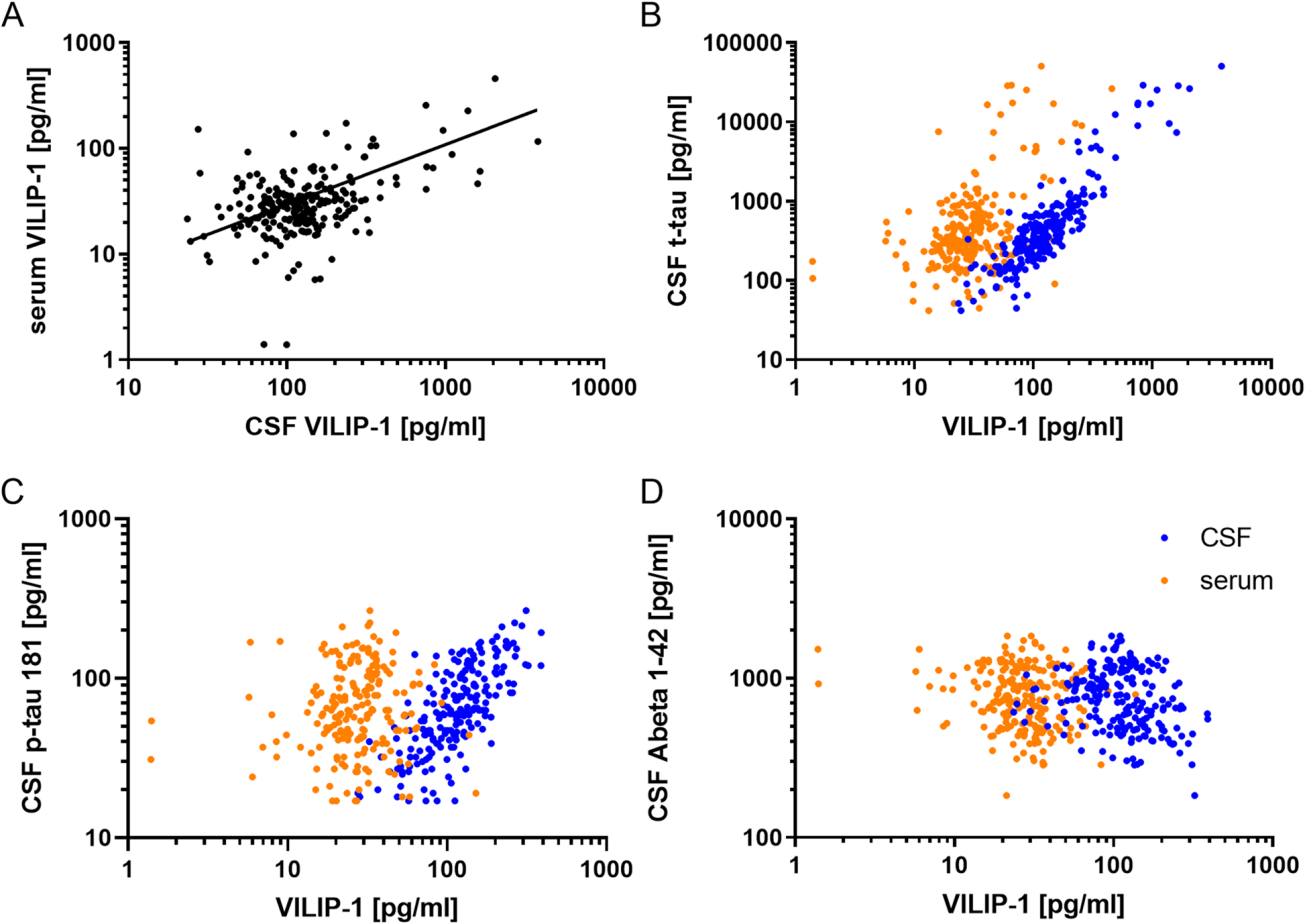
Correlation analysis of CSF and serum VILIP-1 and core AD biomarker. **A** Correlation analysis of CSF and serum VILIP-1 (*r*=0.32 (CI: 0.20–0.43), *p*<0.0001). **B** Correlation analysis of CSF (blue) and serum (orange) VILIP-1 with CSF t-tau (CSF VILIP-1 *r*=0.84 (CI: 0.80–0.87), *p*<0.0001); serum VILIP-1 *r*=0.37 (CI: 0.25–0.048), *p*<0.0001). **C** Correlation analysis of CSF (blue) and serum (orange) VILIP-1 with CSF Abeta 1-42 (CSF VILIP-1 *r*=−0.27 (CI: −0.40 to −0.14), *p*<0.0001; serum VILIP-1 *r*=−0.19 (CI: −0.32 to −0.05), *p*=0.0063). Correlation analysis was performed using Spearman’s rank correlation coefficient

VILIP-1 CSF and serum concentrations correlated weakly with each other (*r*=0.32 (CI: 0.20–0.43), *p*<0.0001) (Fig. 2A). Correlation analysis of CSF and serum VILIP-1 with dementia scores revealed no association for CDR SOB or for MMSE. CSF VILIP-1 levels correlated positively with CSF t-tau (*r*=0.84 (CI: 0.80–0.87), *p*<0.0001) and CSF p-tau 181 (*r*=0.72 (CI: 0.65–0.78, *p*<0.0001) and negatively with CSF Abeta 1-42 (*r*=−0.27 (CI: −0.40 to −0.14), *p*<0.0001). Moreover, serum VILIP-1 also correlated with CSF t-tau (*r*=0.37 (CI: 0.25–0.048), *p*<0.0001) and CSF Abeta 1-42 (*r*=−0.19 (CI: −0.32 to −0.05), *p*<0.01) but not with p-tau 181 (*r*=0.12 (CI: −0.02–0.026), *p*=0.08) (Fig. 2 B, C).

### CSF and serum VILIP-1 as early markers for AD

**Fig. 3:**
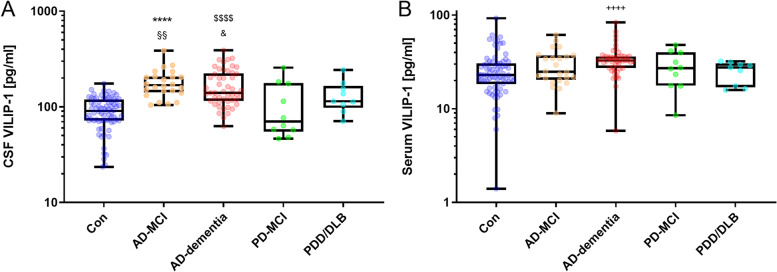
CSF and serum VILIP-1 results in the stratified AD and Parkinson’s syndrome cohort. **A** CSF VILIP-1 results for AD-MCI (*n*=24) and AD-dementia (*n*=49) compared to Con (*n*=75) as well as PD-MCI (*n*=10) and PDD/DLB (*n*=9). ****, *p*<0.0001 vs. Con. §§, *p*=0.0035 vs. PD-MCI. $$$$, *p*<0.0001 vs. Con. &, *p*=0.023 vs. PD-MCI. **B** Serum VILIP-1 results for AD-MCI and AD-dementia compared to Con as well as PD-MCI and PDD/DLB. ++++, *p*<0.0001 vs. Con. Median with IQR for MMSE scores: AD-MCI 25 (22.3–26.8), AD-dementia 20 (15.0–25.0), PD-MCI 28 (26.5–29), and PDD/DLB 22 (19–25). Median with IQR CDR SOB scores: AD-MCI 1.5 (0.5–2.0), AD-dementia 4.5 (3.5–6.5), PD-MCI 0.5 (0–1.1), and PDD/DLB 4.5 (2.5–6). VILIP-1 concentrations are displayed as box plots with single patients shown. Illustrated are the median concentration, the 25% and 75% percentile, and whiskers from minimum to maximum. Groups were compared by the Kruskal-Wallis test and Dunn’s post hoc test. AD-MCI, Alzheimer’s disease with mild cognitive impairment; AD-dementia, Alzheimer’s disease with dementia; Con, non-neurodegenerative control; DLB, dementia with Lewy bodies; IQR, interquartile range; PD-MCI, Parkinson’s disease with mild cognitive impairment; PDD; Parkinson’s disease dementia

For the evaluation of the early diagnostic abilities of VILIP-1, we stratified the AD cases into one cohort of patients with AD-dementia and one with AD-MCI and compared the CSF and serum VILIP-1 level with the Con group. The AD-dementia and AD-MCI groups showed significantly increased CSF VILIP-1 concentrations compared to Con patients (*p*<0.0001) (Fig. 3A). The levels did not differ between the two AD groups. In addition, we stratified the PD spectrum patients into groups with PD-MCI and PDD/DLB. CSF VILIP-1 levels of the AD-MCI (*p*<0.01) and AD-dementia (*p*=0.02) groups were also increased compared to PD-MCI, whereas PD-MCI and PDD/DLB patient levels were not significantly increased compared to Con patients. In the serum analysis, only AD-dementia patients displayed significantly elevated VILIP-1 levels when compared to the Con group (*p*<0.0001).

### Sensitivity and specificity of CSF and serum VILIP-1 for AD diagnosis

**Fig. 4:**
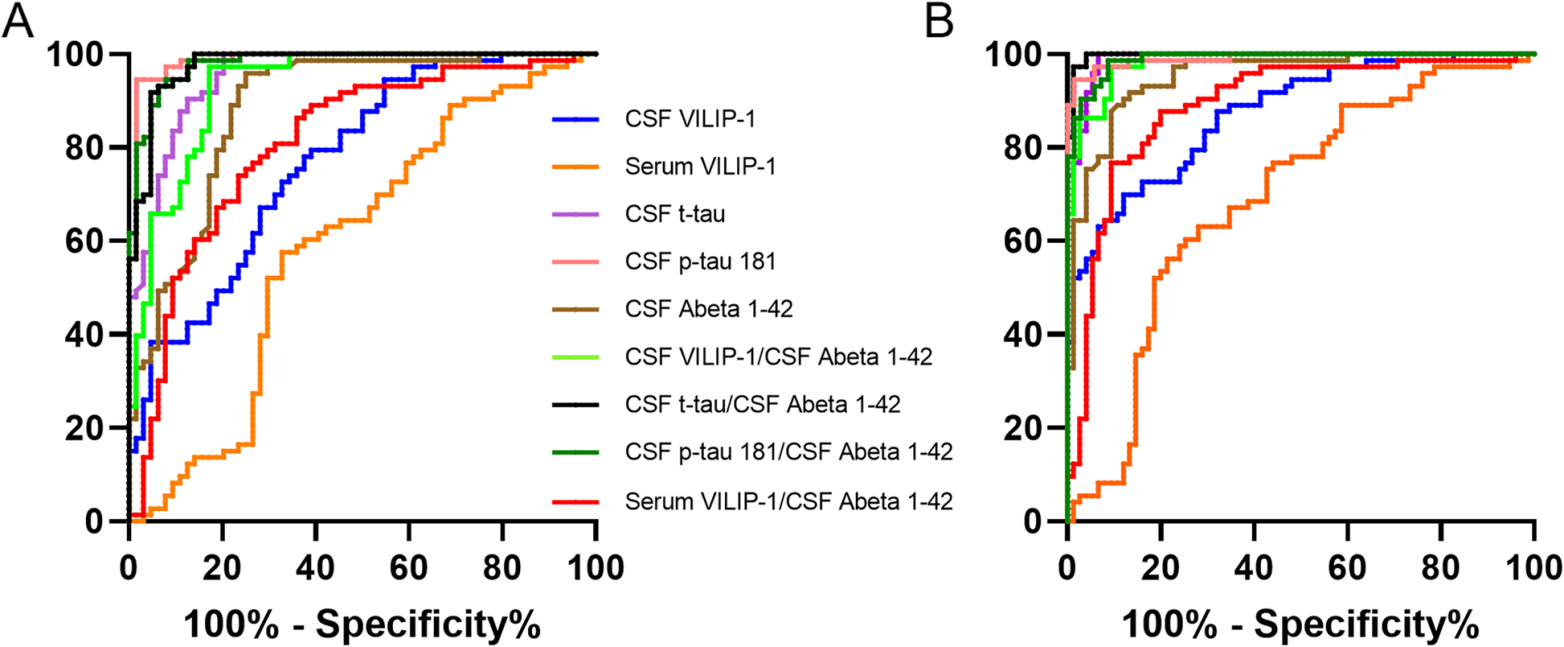
ROC analysis of AD. ROC curves for CSF and serum VILIP-1 as well as core AD biomarkers. **A** Comparison of AD versus all other neurodegenerative diseases except CJD. **B** Comparison of AD versus Con. AD, Alzheimer’s disease; AUC, area under the curve; CJD, Creutzfeldt-Jakob disease; Con, non-neurodegenerative control; ROC, receiver operating characteristics

Using receiver operating characteristic (ROC) analyses, we assessed the discriminating potential of CSF and serum VILIP-1 in the comparison of AD versus the other neurodegenerative diseases with the exception of CJD (Fig. 4A) and AD vs. control patients (Fig. 4B). Moreover, we calculated the Youden index to determine the best cut-off regarding sensitivity and specificity. Additionally, we compared the sensitivity and specificity with the routine markers t-tau, p-tau, and Abeta 1-42 and the ratios of all markers with CSF Abeta 1-42 (Fig. 4 and Table 2).

**Table 2:** Sensitivity and specificity of CSF and serum VILIP-1 for discrimination between AD and the other diagnostic groups except CJD

	AD vs.	Calculated cut-offs	Sensitivity (95% CI) [%]	Specificity (95% CI) [%]	AUCs (95% CI)	Positive likelihood ratio
CSF VILIP-1	ND	>116	78 (67–86)	63 (50–73)	0.77 (0.70–0.85)	2
	Con	>129	70 (59–79)	88 (79–94)	0.87 (0.82–0.93)	6
Serum VILIP-1	ND	>32	58 (46–68)	67 (55–77)	0.59 (0.50–0.69)	2
	Con	>30	63 (52–73)	72 (61–81)	0.69 (0.60–0.77)	2
CSF t-tau	ND	>392	100 (95–100)	80 (68–88)	0.95 (0.92–0.99)	5
	Con	>399	100 (95–100)	93 (85–97)	0.99 (0.98–100)	15
CSF p-tau 181	ND	>94	95 (87–98)	98 (92–99)	0.98 (0.97–1)	59
	Con	>93	95 (87–98)	99 (92–100)	0.99 (0.98–1)	65
CSF Abeta 1-42	ND	<733	96 (89–99)	75 (63–84)	0.88 (0.83–0.94)	4
	Con	<689	92 (83–96)	87 (77–93)	0.95 (0.92–0.98)	7
CSF VILIP-1/CSF Abeta 1-42	ND	>0.174	97 (91–100)	83 (72–90)	0.93 (0.89–0.97)	6
	Con	>0.169	97 (91–100)	91 (82–95)	0.98 (0.97–1)	10
Serum VILIP-1/CSF Abeta 1-42	ND	>0.048	74 (63–83)	77 (65–85)	0.81 (0.74–0.89)	3
	Con	>0.037	88 (78–93)	80 (70–88)	0.89 (0.84–0.95)	4
CSF t-tau/CSF Abeta 1-42	ND	>0.775	92 (83–96)	95 (87–99)	0.98 (0.96–1)	20
	Con	>0.53	100 (95–100)	96 (89–99)	0.99 (0.99–1)	25
CSF p-tau 181/CSF Abeta 1-42	ND	>0.120	93 (85–97)	94 (85–98)	0.98 (0.96–1)	15
	Con	>0.1	99 (93–100)	91 (82–96)	0.99 (0.98–1)	11

Calculated cut-offs in picograms per milliliter for CSF and serum VILIP-1 as well as t-tau, p-tau, and Abeta 1-42. CI, confidence interval; Con, non-neurodegenerative control; ND, non-AD neurodegenerative diseases without CJD

The AUCs of CSF and serum VILIP-1 in the comparison of AD and the other neurodegenerative diseases except CJD were found to be lower with 0.77 (95% 0.70–0.85) and 0.59 (0.5–0.69), respectively, compared to the core markers, e.g., CSF p-tau 0.99 (0.97–1). For the comparison of AD vs. Con, the AUCs of CSF and serum VILIP-1 were calculated as 0.87 (0.82–0.93) and 0.69 (0.60–0.77), respectively, with an increase to 0.98 (0.97–1) and 0.89 (0.84–0.95) when divided by CSF Abeta 1-42 which is comparable with, e.g., the AUC of 0.99 (0.99–1) for the ratio of CSF t-tau and CSF Abeta 1-42. The calculated AUCs of all markers and ratios can be found in Table 2.

## Discussion

In this study, we report on the development and validation of a novel, highly sensitive Simoa assay for the analysis of VILIP-1 in CSF and serum as well as the application of said immunoassay in a cohort of a variety of neurodegenerative diseases. In the performance testing, the assay yielded good results indicating a high sensitivity and reliability of the new quantitative VILIP-1 assay. Applying it to analyze VILIP-1 levels in different neurodegenerative diseases, we could show elevated CSF VILIP-1 concentrations in AD compared to Con patients confirming previous findings [20, 33, 34]. Furthermore, we found a significant increase in AD CSF VILIP-1 levels compared to PD spectrum, bvFTD, and ALS corroborating data from a study which showed elevated CSF VILIP-1 levels in AD compared to patients with DLB [35] as well as a study which depicted increased CSF VILIP-1 AD levels compared to non-AD-dementias (FTD (*n*=11), PSP (*n*=7), LBD (*n*=1)) [20]. Up to now, only one study compared AD CSF VILIP-1 levels with a separate FTD cohort and Lewy body disease (LBD) patient concentrations also detecting a significant increase in AD levels compared to LBD but not to FTD [19]. One possible explanation for the discrepancy could be the highly heterogeneous disease group of FTDs rendering it more difficult to compare between studies. Whereas ALS VILIP-1 concentrations remain on a level with Con, CJD patients displayed by far the highest VILIP-1 levels of all analyzed groups. Because of the high correlation between tau protein levels and VILIP-1, this result might have been expected. Further studies have to be performed to explore the potential of VILIP-1 as a biomarker in CJD in more detail.

Our findings show a high correlation of CSF VILIP-1 with t-tau and p-tau and a weaker, inverse correlation with Abeta 1-42 which supports earlier studies [11, 20]. Moreover, we could confirm findings that CSF VILIP-1 is already increased in patients with mild cognitive impairment due to AD grouped by CDR-SOB and could therefore be a valuable marker for the early diagnosis of AD [11, 19, 20]. Additionally, we could show that the elevation in MCI patients is not specific for all MCI patients as PD-MCI did not display increased levels. The PD-MCI group, however, is small in number and future studies with more patients need to confirm these findings.

Blood VILIP-1 concentrations in the serum/plasma of AD patients have only been analyzed in one study [20]. In 2011, Tarawneh et al. detected a significant, but in relation to CSF smaller difference between VILIP-1 levels in AD compared to cognitively normal control patients in plasma. Studies on blood VILIP-1 concentrations in other neurodegenerative diseases are completely missing. Our findings on serum VILIP-1 in AD patients showed increased concentrations compared to the Con group but the difference between the two groups was smaller than for CSF confirming the plasma measurements from Tarawneh et al. Furthermore, the significant difference between AD and PD spectrum, bvFTD, and ALS seen in CSF was not found for blood. The main reason seems to be the higher variability of VILIP-1 levels in serum compared to CSF which might be explained by the expression pattern of VILIP-1 which is mainly but not exclusively synthesized in the brain. VILIP-1 has for example been shown to be present in the liver, lung, heart, and testis [36]. VILIP-1 proteins from these organs will eventually end up in the blood stream. Here, they mix with VILIP-1 molecules from the CSF, thereby masking, in the blood stream, the ongoing processes in the brain represented by the CSF levels. In fact, this hypothesis is also supported by our data regarding the very weak correlation of CSF and blood VILIP-1 concentrations. As a result, single blood VILIP-1 measurements alone seem not to be of high clinical relevance in the diagnosis of AD. However, the analysis of serum VILIP-1 could still be useful, as a low-invasive method in follow-up investigations. In contrast, in CJD, the difference to the other diseases remains which renders blood VILIP-1 an easy accessible possible marker for neuronal injury in CJD.

Serum VILIP-1 levels were significantly elevated in AD-dementia compared to Con but neither to any other neurodegenerative disease nor to AD-MCI patients. Furthermore, serum VILIP-1 levels of the AD-MCI cohort displayed no significant difference to the Con group as observed for CSF VILIP-1 levels. One possible explanation could be differences in the blood-cerebrospinal fluid barrier (BCB) dysfunction between the AD-dementia and AD-MCI groups. In fact, BCB impairment has been described in the pathogenesis of AD; however, studies so far could not reveal a significant correlation between cognitive function and BCB impairment [37, 38]. Another reason for the differences between serum and CSF VILIP-1 levels could be a potential bias constituted by the lower number of AD-MCI compared to the AD-dementia patients. To be sure of the role of serum VILIP-1 in the early diagnosis of AD extended analyses concentrating on this aspect, as has been done for CSF VILIP-1, with higher AD-MCI patient numbers and follow-up sampling are needed.

The difference between CSF and blood VILIP-1 results is also reflected in the ROC analysis where with an AUC of 0.77 CSF VILIP-1 performs all right in the differential diagnosis of AD and well when compared to the Con group (AUC 0.87). Serum VILIP-1 on the other side shows only an AUC of 0.59 versus the other neurodegenerative diseases and an AUC of 0.69 versus the Con group. Applying the ratio CSF VILIP-1 to CSF Abeta 1-42 in the comparison with the other neurodegenerative diseases, the AUC increases substantially to 0.93 which is close to the AUC of CSF t-tau/ CSF Abeta 1-42 (0.98). These findings suggest that future analysis especially also the combination of blood VILIP-1 with blood Abeta 1-42, for which more and more measurement techniques emerge [39–41], could yield better discriminating results.

Another neuronal injury marker described in the literature is neurofilaments. Especially, neurofilament light (NfL) and heavy chain have been extensively studied as markers in neurological disorders [42]. CSF and blood NfL levels have been shown to be elevated in AD compared to control patients [43–45] but also in FTD [43, 46] and especially in ALS patients [47–49] which renders VILIP-1 compared to neurofilaments the more specific marker for neuronal injury in the tested neurodegenerative disease groups. An explanation could be a specific vulnerability of VILIP-1 synthesizing neurons to calcium-mediated neurodegeneration in AD pathophysiology [50]. In addition, VILIP-1 concentrations did not or only weakly correlate with age in contrast to CSF and blood NfL levels which have been shown to be strongly associated with age [42].

A biomarker yielding high specificity for AD, however, representing a different pathology in AD than VILIP-1 is p-tau [51]. As our findings demonstrate, VILIP-1 levels are inferior when measured in CSF or blood in the discrimination between AD and other neurodegenerative diseases compared to the high specificity of p-tau. Nevertheless, VILIP-1 as a biomarker for AD has its strengths. The main one is the reflection of different aspects of AD pathology than the core ATN markers. As a neuronal calcium-sensor protein, it has an important role in the disturbed calcium ion balance associated with AD. Hence, VILIP-1 could be a possible marker for monitoring of calcium homeostasis and be used as an additional readout in clinical trials, e.g., of the effect of therapeutic agents on the calcium ion balance. Furthermore, VILIP-1 is involved in the regulation of synaptic plasticity [11] and could therefore possibly also be a readout for synaptic (dys)function in AD patients. Future follow-up studies will have to confirm this by correlating VILIP-1 level changes with scores for cognitive function over time. Moreover, emerging biomarkers like VILIP-1 could potentially substitute for t-tau and p-tau as markers of AD brain pathology in studies where tau-related biomarkers may be directly affected (e.g., drug trials of anti-tau antibodies), similarly for Abeta.

### Limitations

The strengths of our study are (i) the establishment of a novel, easy-to-use Simoa assay for the analysis of CSF and serum VILIP-1, (ii) the outline of a comprehensive study of CSF and serum VILIP-1 concentrations in a cohort of different neurodegenerative diseases, and (iii) the parallel analysis of CSF and serum samples in all cohorts. Limitations of the study are the lower number of AD-MCI compared to AD-dementia cases and the lack of a vascular dementia (VaD) control cohort.

## Conclusions

In the clinic, VILIP-1 analysis could be in addition relevant for patient stratification for clinical and as an additional readout in therapeutic trials for example for monitoring the effect of disease-modifying drugs on calcium ion homeostasis or synaptic/neuronal integrity. Further studies might focus on the analysis of VILIP-1 in VaD, on the combination of markers in blood, and especially on the investigation of blood VILIP-1 levels in follow-up samples.

## Supplementary Information

**Additional file 1: Supplementary material.** Description of assay parallelism and pre-analytical stability.

## Data Availability

All data generated or analyzed during this study are included in this published article [and its supplementary information files].

## Abbreviations

AD: Alzheimer’s disease
ALS: Amyotrophic lateral sclerosis
bvFTD: Behavioral variant frontotemporal dementia
CDR: Clinical dementia rating
CJD: Creutzfeldt-Jakob disease
Con: Non-neurodegenerative controls
CSF: Cerebrospinal fluid
DLB: Dementia with Lewy bodies
FTD: Frontotemporal dementia
MCI: Mild cognitive impairment
MMSE: Mini-Mental State Examination
NfL: Neurofilament light chain
PD: Parkinson’s disease
PDD: Parkinson’s disease dementia
VILIP-1: Visinin-like protein 1
