# Supplementary material for: Visinin-like protein 1 levels in blood and CSF as emerging markers for Alzheimer’s and other neurodegenerative diseases
Source: Alzheimers Res Ther. 2022 Nov 22;14:175. doi: 10.1186/s13195-022-01122-4 (PMC9682835; doi:10.1186/s13195-022-01122-4)
Supplement: Supplementary file 1 — Additional file 1: Supplementary material. Description of assay parallelism and pre-analytical stability. [file 13195_2022_1122_MOESM1_ESM.pdf]

Visinin-like protein 1 levels in blood and CSF as emerging markers for Alzheimer's and other neurodegenerative diseases

Supplementary material

Steffen Halbgebauer, PhD[1], Petra Steinacker, PhD[1, 5], Daniel Riedel[1], Patrick Oeckl, PhD[1, 4], Sarah Anderl-Straub, PhD[1], Jolina Lombardi, PhD[1], Christine A.F. von Arnim, MD[1, 2], Magdalena Nagl, PhD[1], Armin Giese, MD[3], Albert C. Ludolph, MD[1, 4], Markus Otto, MD[1,5]

1. Department of Neurology, Ulm University Hospital, Oberer Eselsberg 45, 89081 Ulm, Germany.

2. Division of Geriatrics, University Medical Center Göttingen, Göttingen, Germany.

3. Department of Neuropathology, Ludwig-Maximilians-University, Munich, Germany.

4. Deutsches Zentrum für Neurodegenerative Erkrankungen (DZNE e.V.), Ulm, Germany.

5. Department of Neurology, Halle University Hospital, Martin Luther University Halle/Wittenberg, Ernst-Grube Strasse 49, 06120 Halle (Saale), Germany.

Corresponding Author contact information:

Prof. Markus Otto, MD, Department of Neurology, University of Ulm, Oberer Eselsberg 45, 89081 Ulm, Germany. E-mail: markus.otto@uni-ulm.de, Tel.: +4973150063010, Fax: +4973150063002

and

Department of Neurology, University clinic, Martin Luther University, Ernst-Grube Strasse 40, 06120 Halle (Saale), Germany. E-mail: markus.otto@uk-halle.de, Tel.: +49 345- 557 2858, Fax: 0345 557 2860

Assay parallelism

Multiple CSF and serum specimen were prepared in a dilution series in order to investigate assay parallelism. The CVs for the different diluted CSF samples were determined to be below 15 % except for the neat CSF specimen. Back-calculated CSF VILIP-1 levels were calculated to be within the range of 83 % - 105 % in comparison to the corresponding CSF sample which was diluted in a ratio 1:4. In the neat CSF sample, 46 % of the mean back-calculated VILIP-1 levels compared to the corresponding CSF sample diluted 1:4 could be detected. Similarly, in diluted serum specimen CVs for VILIP-1 levels were determined to be below 15 %. Besides, back-calculated VILIP-1 levels were determined to be in the range of 90 % – 110 % compared to the corresponding serum specimen which was diluted in a ratio 1:4 (Fig. S1).

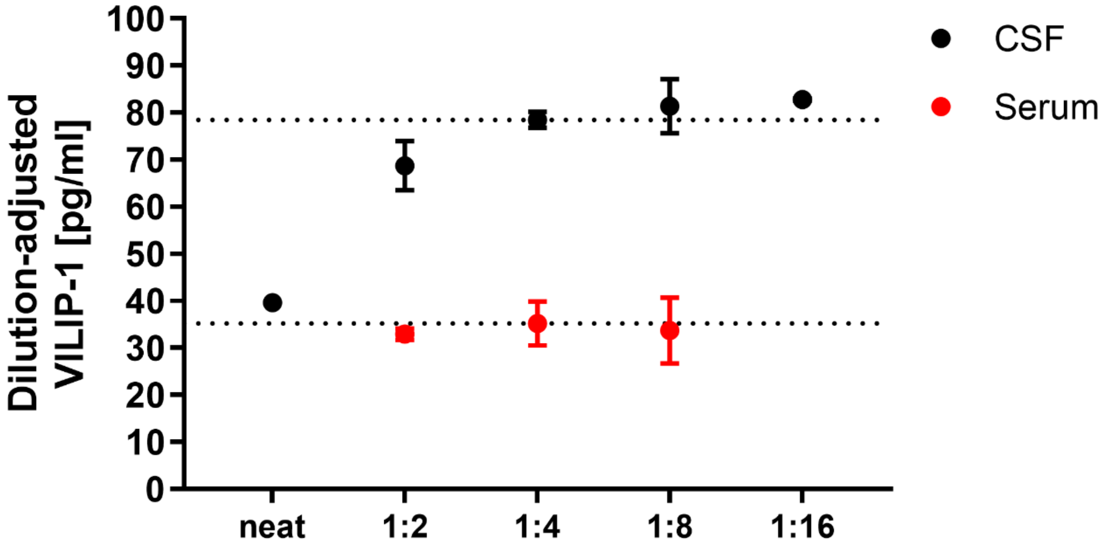

Figure S1. Assay parallelism.

Endogenous VILIP 1 concentrations were analyzed in CSF and serum samples. Black circles depict CSF and red circles results for serum samples. Results are reported as mean dilution-adjusted VILIP 1 concentrations ± standard deviation. CSF, cerebrospinal fluid; VILIP 1 Visinin-like protein 1.

Pre-analytical stability of CSF and serum VILIP-1

In order to investigate the pre-analytical stability of CSF and serum VILIP-1 multiple independent serum and CSF samples were subjected to experimental freeze/thaw and storage conditions. After four experimental freeze/thaw cycles serum VILIP-1 concentrations were determined to be within 82 % and 115 % of the reference value (Fig. S2C). CSF VILIP-1 specimens were assessed for two freeze/thaw cycles only. After two experimental freeze/thaw cycles the CSF VILIP-1 levels were found to be within the range of 97 % and 102 % of the reference value (Fig. S2A). When assessing storage stability over 120 h storage at room temperature, serum VILIP-1 concentrations were found to be in the range between 97 % and 110 % of the reference values (Fig. S2D) and CSF VILIP-1 concentrations were found at 90 % of the reference sample (Fig. S2B). Similarly, when storage stability was investigated over 120 h storage at 4 °C, serum VILIP-1 concentrations were determined to be in the range of 107 % and 109 % of the reference values (Fig. S2D) and CSF VILIP-1 levels were found to be at 113 % of the reference samples (Fig. S2B).

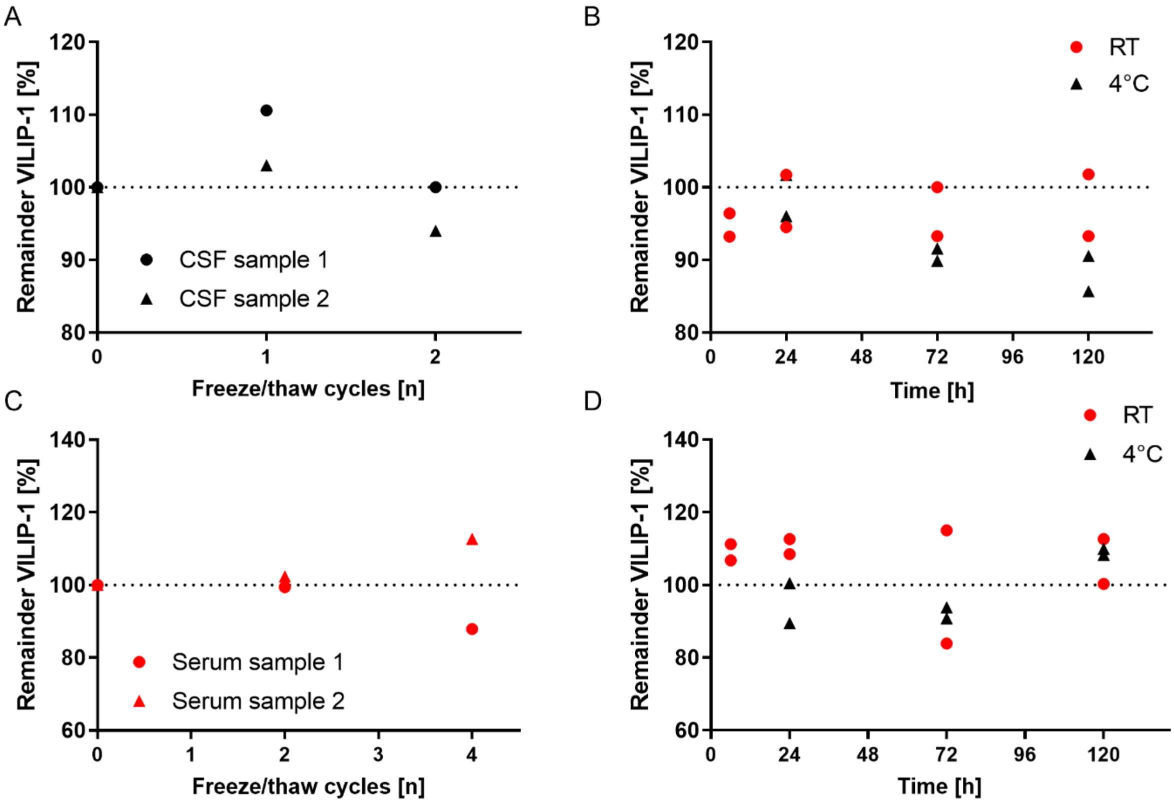

Figure S2. Determination of stability of CSF and serum VILIP-1.

(A) CSF VILIP-1 results for experimental freeze/thaw cycles. (B) CSF VILIP-1 results for different experimental storage stability conditions. Two independent CSF samples were analyzed. (C) Serum VILIP-1 results for experimental freeze/thaw cycles. (D) Serum VILIP 1 results for experimental storage stability conditions. Two independent serum samples were analyzed. Circles depict RT and triangles 4 °C results. Symbols represent mean VILIP-1 levels from duplicate measurement, normalized to the reference sample. RT, room temperature; VILIP-1, Visinin-like protein 1
